# Bacteriological profiles, antimicrobial resistance patterns, and predictors of culture-confirmed neonatal Sepsis at Asella Teaching Hospital, Southeast Ethiopia

**DOI:** 10.1371/journal.pone.0345245

**Published:** 2026-03-25

**Authors:** Mesfin Wubishet Gurmu, Samuel Manahle, Tesfa G/Meskel, Solomon Gelaye Yinges, Gebi Agero, Liyat Kebede

**Affiliations:** 1 Department of Pediatrics, Arsi University College of Health Sciences, Asella, Ethiopia; 2 Department of Public Health, Arsi University College of Health Sciences, Asella, Ethiopia; Debre Markos University, ETHIOPIA

## Abstract

**Background:**

Neonatal sepsis remains a major cause of morbidity and mortality in low- and middle-income countries. Empirical antibiotic therapy is commonly initiated before culture results are available, in accordance with World Health Organization (WHO) recommendations; however, its effectiveness depends on local pathogen distribution and antimicrobial resistance patterns, which vary across healthcare settings. This study aimed to determine the bacteriological profile, antimicrobial susceptibility patterns, and predictors of culture-confirmed neonatal sepsis at Asella Teaching Hospital in Southeast Ethiopia.

**Methods:**

A retrospective cross-sectional study was conducted among 392 neonates with clinically suspected sepsis who were admitted to the NICU between January 2021 and December 2023. Participants were selected using systematic random sampling from medical registry records. Blood cultures and bacterial identification were performed using standard microbiological procedures. Antimicrobial susceptibility testing was conducted using the Kirby–Bauer disk diffusion method in accordance with CLSI 2021 guidelines. Multivariable logistic regression analysis was used to identify independent predictors of culture-confirmed sepsis, with statistical significance set at *p* < 0.05.

**Results:**

Culture-confirmed sepsis was identified in 246 neonates (62.8%; 95% CI: 57.9–67.1), with early-onset sepsis accounting for 71.9% of cases. Gram-positive (50.8%) and Gram-negative (49.2%) organisms were nearly equally distributed. Among Gram-positive isolates, coagulase-negative staphylococci (35%) and *Staphylococcus aureus* (8.1%) were predominant, whereas *Klebsiella pneumoniae* (22.4%) and *Acinetobacter* spp. (8.5%) were the leading Gram-negative pathogens. Gram-positive isolates showed the highest susceptibility to vancomycin (91.9%), followed by trimethoprim–sulfamethoxazole (76.3%) and amikacin (72.9%), while high resistance was observed to ampicillin (91.9%) and ciprofloxacin (80.5%). Gram-negative isolates were most susceptible to amikacin (85.8%) and carbapenems (75.8%), but demonstrated substantial resistance to ampicillin (92.5%) and third-generation cephalosporins (65.8–93.3%). Multivariable logistic regression analysis identified several independent predictors of culture-confirmed neonatal sepsis, including preterm birth (AOR = 2.20; 95% CI: 1.07–4.98), tachypnea at admission (AOR = 4.79; 95% CI: 2.73–8.41), hypothermia (AOR = 2.35; 95% CI: 1.12–4.92), prolonged hospitalization (AOR = 2.51; 95% CI: 1.52–4.16), maternal chorioamnionitis (AOR = 4.49; 95% CI: 2.35–8.80), and a low fifth-minute Apgar score (AOR = 3.95; 95% CI: 1.87–8.34).

**Conclusion:**

Culture-confirmed neonatal sepsis was highly prevalent, with a substantial burden of multidrug-resistant pathogens. Most bacterial isolates exhibited high resistance to commonly used first-line antibiotics, whereas amikacin and carbapenems remained relatively effective. The high prevalence of multidrug-resistant Gram-negative bacteremia is particularly concerning and underscores the need for routine antimicrobial resistance surveillance, locally guided empirical antibiotic therapy, strengthened infection prevention and control measures, and robust antimicrobial stewardship programs to reduce the emergence of antimicrobial resistance and improve neonatal outcomes in this setting.

## Introduction

Neonatal sepsis is a life-threatening systemic infection occurring during the first 28 days of life and remains a major contributor to neonatal morbidity and mortality worldwide. The condition often presents with nonspecific clinical manifestations such as temperature instability, respiratory distress, poor feeding, and lethargy, which complicates early diagnosis, particularly in resource-limited settings where advanced diagnostic tools are scarce [1,2]. Globally, neonatal sepsis accounts for a substantial proportion of neonatal deaths, with an estimated incidence exceeding 2,200 cases per 100,000 live births and case-fatality rates ranging from 11% to 19% [3]. The burden is disproportionately high in low- and middle-income countries, particularly in sub-Saharan Africa, where infectious diseases remain a leading cause of neonatal mortality [4,5]. In Ethiopia, the reported prevalence of neonatal sepsis among hospitalized neonates ranges from approximately 45% to 78%, reflecting significant regional variation in disease burden and healthcare capacity [6–9].

Neonatal sepsis is typically classified as early-onset sepsis (EONS) or late-onset sepsis (LONS) based on the timing of symptom onset. EONS occurs within the first 72 hours of life and is often associated with vertical transmission of pathogens from the maternal genital tract during labor or delivery. In contrast, LONS develops after 72 hours and is usually related to environmental exposure or healthcare-associated infections [10,11]. The spectrum of causative pathogens varies geographically and temporally. In high-income countries, *Group B Streptococcus* and *Escherichia coli* are the most common etiological agents, whereas studies from many African countries report a predominance of Gram-negative bacteria, particularly *Klebsiella pneumoniae*, *Acinetobacter* species, and *Enterobacter* species, alongside Gram-positive pathogens including CoNS and *S. aureus* [10–14]. Recent evidence from low- and middle-income countries highlights a growing predominance of multidrug-resistant (MDR) Gram-negative bacteria, especially extended-spectrum beta-lactamase (ESBL)-producing *Enterobacteriaceae* and *Acinetobacter* species, even among early-onset cases. Additionally, a high prevalence of methicillin-resistant *Staphylococcus aureus* (MRSA) has been reported [13–17].

Diagnosis often relies on blood cultures, but in practice, empiric antibiotic therapy usually begins before culture results are available, particularly where laboratory capacity is limited [2,18]. The World Health Organization recommends a combination of ampicillin (or benzylpenicillin) and gentamicin as first-line empirical treatment in resource-limited settings [18]. However, the widespread use of empirical antibiotics has contributed to the emergence of antimicrobial resistance (AMR), including multidrug-resistant organisms such as methicillin-resistant *Staphylococcus aureus* (MRSA) and extended-spectrum beta-lactamase (ESBL)–producing Gram-negative bacteria. The increasing prevalence of resistant pathogens poses a major challenge to neonatal care and threatens the effectiveness of standard treatment regimens [16,17,19–21]. Resistant pathogens are estimated to contribute to approximately 214,000 neonatal deaths annually, further complicating sepsis management [19].

Because pathogen distribution and resistance patterns vary across regions and healthcare facilities, local surveillance data are essential for guiding empirical therapy and antimicrobial stewardship efforts [20,22]. In Ethiopia, several studies have documented increasing resistance to commonly used antibiotics such as ampicillin and third-generation cephalosporins [23–25]. However, limited data are available from Southeast Ethiopia, particularly from tertiary referral hospitals that manage a high burden of critically ill neonates. Therefore, this study aimed to determine the prevalence of culture-confirmed neonatal sepsis, characterize the bacteriological profile, assess antimicrobial susceptibility patterns, and identify predictors of culture-confirmed bacteremia among neonates admitted to the neonatal intensive care unit of Asella Teaching Hospital. The findings are expected to inform local antibiogram development and clinical management guidelines, thereby supporting evidence-based treatment and preventive strategies to improve neonatal outcomes.

## Methods and materials

### Study design, setting, and period

An institution-based retrospective cross-sectional study was conducted at the neonatal intensive care unit (NICU) of Asella Referral and Teaching Hospital (ARTH) in Asella town, Southeast Ethiopia, located approximately 175 km south of Addis Ababa. ARTH serves as a referral center for an estimated four million people from both urban and rural communities. The hospital has a dedicated neonatal unit staffed by pediatric specialists and trained neonatal nurses experienced in managing neonatal sepsis. It provides comprehensive diagnostic services, including medical microbiology, clinical chemistry, hematology, serology, and parasitology. The microbiology laboratory is equipped to process clinical specimens, perform microbial culture and identification, and conduct antimicrobial susceptibility testing (AST). This study reviewed medical records of neonates admitted to the NICU between January 1, 2021, and December 30, 2023.

### Source and study population

All neonates who were admitted to the NICU of ARTH were the source population. The study population included neonates aged 0–28 days who were admitted to the NICU with clinically suspected sepsis and had documented blood culture results during the study period.

### Eligibility criteria

**Inclusion criteria:** All neonates with a presumptive diagnosis of neonatal sepsis, as determined by the attending clinician, were included if they had documented blood culture results and complete clinical and laboratory records, including antimicrobial susceptibility testing (AST) performed according to CLSI guidelines.

**Exclusion criteria:** Neonates were excluded if they had received antibiotics before admission, had critical congenital anomalies, or had incomplete medical records. Additionally, cases with contaminated blood cultures or bacterial growth without accompanying AST data were excluded from the study.

### Sample size determination and sampling technique

The sample size was calculated using both the single population proportion and double population proportion formulas, based on previously reported prevalence estimates of bacterial isolates and antimicrobial resistance (AMR) patterns in comparable settings. For estimating the prevalence of bacterial isolates, the single population proportion formula was applied using a 36% prevalence of *E. coli* reported in a study from Northeastern Ethiopia [26]. With a 95% confidence level (α = 0.05; Z = 1.96) and a 5% margin of error, the calculated sample size was 355. To assess factors associated with culture-confirmed sepsis, including age, birth weight, and gestational age, the double population proportion formula with power analysis was applied using Epi Info statistical software. The maximum sample size from this analysis, based on age >3 days, was 289.


$$ni=\;\frac{{\left(\frac{Z\propto }{\text{2}}\right)}^{\text{2}}\;*p(\text{1}-p)}{d^{\text{2}}}\text{ n}=\;\frac{\left(\text{1}.\text{9}\text{6}{)}^{\text{2}}\;(\text{0}.\text{3}\text{6})(\text{1}-\text{0}.\text{3}\text{6}\right)}{{\left(\text{0}.\text{0}\text{5}\right)}^{\text{2}}}=\text{3}\text{5}\text{5}$$

Considering all calculations, the largest sample size (n = 355) was selected to ensure adequate statistical power. After adding 10% to account for incomplete or inaccessible records, the final sample size was set at 392. Since this was smaller than the total number of neonates admitted with suspected sepsis during the study period (N = 2,196), systematic random sampling was employed. A sampling interval (k) of 6 was determined by dividing the total eligible population by the required sample size, and every sixth neonate was selected from the admission registry logbook.

### Data collection procedure

Data were collected using a structured checklist developed in Open Data Kit (ODK) to ensure standardized and systematic data capture. Clinical, demographic, and laboratory information were extracted from neonatal medical records using this predefined tool. Blood culture results and antimicrobial susceptibility data were obtained from the Bacteriology Unit of the ARTH Laboratory Department. Eligible neonates were identified via electronic medical record numbers from the microbiology registry, and each neonate was included only once to maintain data integrity and prevent duplication. The checklist captured key neonatal characteristics, including age, sex, gestational age, birth weight, mode of delivery, and relevant clinical risk factors. Additional information included NICU admission status, date of admission, presenting clinical features, and prior antibiotic exposure. Maternal variables, such as gestational age at delivery, mode of delivery, and history of prolonged rupture of membranes, were also recorded. Blood culture findings and antimicrobial susceptibility patterns were systematically documented. For antimicrobial resistance analysis, isolates reported as having intermediate susceptibility were classified as resistant to facilitate clearer interpretation of resistance trends.

## Laboratory methods

### Blood culture and bacterial identification

A standardized protocol was followed for blood collection, culture, and bacterial identification. Blood culture bottles were incubated at 35–37 °C under aerobic conditions and monitored daily for up to seven days for bacterial growth. Bottles were inspected macroscopically for indicators of growth, including hemolysis, turbidity, and gas production. Positive broths were sub-cultured onto blood agar, chocolate agar, MacConkey agar, and mannitol salt agar, followed by incubation at 35–37 °C for 24 hours. This subculturing process was repeated until day seven, after which blood cultures without growth were considered negative. Bacterial identification was based on colony morphology, Gram staining, and standard biochemical tests. Gram-negative bacteria were further characterized using indole, urease, lysine decarboxylase, triple sugar iron agar, citrate utilization, oxidase, and motility tests. Gram-positive bacteria were identified using catalase and coagulase tests, along with hemolytic patterns, in accordance with the Clinical and Laboratory Standards Institute (CLSI) 2021 guidelines [27].

### Antimicrobial susceptibility testing

Antimicrobial susceptibility testing was performed for each isolate using the disc diffusion method on Mueller-Hinton agar, following CLSI 2021 guidelines [27]. For fastidious organisms, Mueller-Hinton agar supplemented with 5% sheep blood was used. Three to five pure colonies of each isolate were emulsified in 2 mL of sterile normal saline. The turbidity of the suspension was adjusted to a 0.5 McFarland standard (~5% turbidity). A sterile cotton swab was dipped into the suspension, and excess fluid was removed by pressing against the tube wall. The bacterial suspension was then uniformly streaked onto the surface of the appropriate agar medium: Mueller-Hinton agar for non-fastidious organisms and Mueller-Hinton agar with 5% sheep blood for fastidious organisms. Antibiotic-impregnated discs were placed on the agar surface, allowing diffusion of the antimicrobial agents, and the plates were incubated at 37 °C for 24 hours. The antibiotics tested included ampicillin, gentamicin, ceftriaxone, cefepime, amikacin, meropenem, imipenem, vancomycin, trimethoprim-sulfamethoxazole, clindamycin, and others according to hospital protocol. The diameters of the inhibition zones surrounding each disc were measured, and isolates were classified as sensitive, intermediate, or resistant, based on CLSI 2021 standards [27]. Multidrug resistance (MDR) was defined as resistance to ≥1 agent in ≥3 antimicrobial categories [28].

### Study variables

Dependent variable: Culture-confirmed neonatal sepsis.

Independent variable

- **Maternal factors:** educational status, gravidity and parity, ANC follow-up, UTI, duration of labor, chorioamnionitis, and antibiotic treatment.
- **Neonatal factors:** age, sex, gestational age, birth weight, and multiple births.
- **Health care-related factors:** place of delivery, mode of delivery, resuscitation, neonatal feeding practice, length of hospital stay, and blood transfusion.

### Operational and case definitions

**Clinical (suspected) sepsis (CS):** Clinical (suspected) sepsis was defined as a diagnosis made by a clinician based on the presence of signs and symptoms suggestive of neonatal sepsis, with or without associated maternal or neonatal risk factors, in accordance with the Integrated Management of Neonatal and Childhood Illness (IMNCI) guideline [29]. Neonatal sepsis was considered in neonates aged ≤28 days presenting with one or more of the following: temperature instability (fever ≥38°C or hypothermia ≤36°C), hemodynamic instability, convulsions, lethargy, feeding intolerance, hypoglycemia, vomiting, bulging fontanelle, respiratory distress, apnea, jaundice, skin signs of infection, or umbilical pus discharge or hyperemia.

**Culture-confirmed sepsis (CPS):** Neonates with clinical features of sepsis and laboratory evidence supportive of infection, accompanied by a positive blood culture demonstrating a bacterial pathogen [30].

**Antimicrobial susceptibility pattern:** The classification of bacterial isolates as susceptible, intermediate, or resistant to specific antibiotics based on inhibition zone diameters. For analytical purposes, isolates categorized as “intermediate” were grouped with “resistant.”

**Multidrug-Resistant Organism (MDRO):** A microorganism exhibiting resistance to at least one antimicrobial agent in three or more distinct classes of antibiotics [28].

**Narrow-spectrum antibiotics:** Defined as antibiotics with limited antimicrobial coverage, including ampicillin, gentamicin, cloxacillin, and amikacin.

**Broad-spectrum antibiotics:** Defined as antibiotics with extended antimicrobial coverage not included in the narrow-spectrum group; commonly used agents include vancomycin, meropenem, and cefotaxime.

**Early-onset sepsis (EONS):** Sepsis occurring within the first 72 hours of life, typically resulting from vertically transmitted maternal pathogens [30].

**Late-onset sepsis (LONS):** Sepsis occurring after 72 hours of life, generally associated with horizontally acquired pathogens [30].

**First-line antibiotics**: Ampicillin and gentamicin [31].

**Second-line antibiotics:** Third-generation cephalosporins [1,31].

**Third-line antibiotics:** Includes amikacin, piperacillin–tazobactam, cefepime, vancomycin, and ciprofloxacin [1,31].

### Data quality assurance

Standard operating procedures (SOPs) for microbiological techniques were strictly followed during blood sample collection, transportation, inoculation onto culture media, incubation, and biochemical identification. The sterility of prepared culture media was verified by randomly selecting and incubating 5% of each production batch. Media performance was routinely evaluated using standard reference strains: *Escherichia coli* (ATCC 25922), *Staphylococcus aureus* (ATCC 25923), and *Pseudomonas aeruginosa* (ATCC 27853). All procedures related to culture inoculation, colony morphology assessment, measurement, and interpretation of antimicrobial susceptibility testing were performed and monitored by experienced microbiology professionals to ensure accuracy, reliability, and consistency. A standardized data extraction checklist was developed following a systematic review of relevant literature [26,32–35]. The checklist was pretested on 5% of the total sample size (n = 20) using randomly selected medical records prior to the commencement of data collection. The pretest assessed clarity, completeness, consistency, and feasibility. Based on the findings, necessary modifications were made, including the addition of previously omitted antibiotics relevant to susceptibility testing. Data were collected by four trained personnel (two medical interns and two laboratory technologists). All data collectors received one day of standardized training prior to data collection. The data collection process was closely supervised by the principal investigator, who reviewed completed forms daily for completeness, consistency, and accuracy before data entry.

### Data processing and statistical analysis

Collected data were checked for completeness and consistency prior to entry. Data were entered into Microsoft Excel for initial cleaning, including verification of missing values, duplicate entries, and outliers. The cleaned dataset was subsequently exported to SPSS version 27 for statistical analysis. Continuous variables were summarized using means and standard deviations, whereas categorical variables were presented as frequencies and percentages. Cross-tabulations were performed where appropriate. Binary logistic regression analysis was conducted to assess the association between independent variables and culture-confirmed neonatal sepsis. Variables with a p-value < 0.25 in the univariate analysis were included in the multivariate logistic regression model. Adjusted odds ratios (AORs) with 95% confidence intervals (CIs) were calculated. Statistical significance was determined at a p-value < 0.05.

### Ethical consideration

Ethical approval was obtained from the Ethical Review Committee of Arsi University, College of Health Sciences (Protocol No. A/CHS/RC/104/2024), and permission to access hospital data was granted by the hospital administration. The study was conducted in accordance with institutional guidelines, national research regulations, and the Declaration of Helsinki. As the study used retrospective secondary data, informed consent was waived by the Institutional Review Board. The dataset was accessed on November 20, 2024. All data were fully anonymized before access, and confidentiality was strictly maintained, with access limited to the principal investigator.

## Results

### Background and clinical characteristics of the study participants

A total of 392 neonates with clinically diagnosed sepsis were included in the study. The majority (319; 81.4%) were ≤7 days old at presentation, and 255 (65.1%) were male. Regarding gestational age, 173 neonates (44.1%) were preterm, while 219 (55.9%) were term. Low birth weight was documented in 163 cases (41.6%). Early-onset sepsis (EOS) accounted for most presentations (282; 71.9%). Half of the neonates (200; 51.0%) required hospitalization for ≥7 days. Prolonged rupture of membranes occurred in 74 deliveries (18.9%), and prolonged labor was reported in 21 cases (5.4%). Multiple gestations accounted for 59 births (15.0%). At admission, tachypnea was observed in 294 neonates (75.0%). Nearly all mothers (385; 98.4%) had attended antenatal care (ANC). Intrapartum fever was documented in 37 mothers (9.5%), clinical chorioamnionitis in 98 (25.0%), and UTI/STI in 23 cases (5.9%). Intrapartum antibiotic prophylaxis was administered to 100 mothers (25.5%) (Table 1).

**Table 1:** Background and Clinical Characteristics of Neonates with Suspected Septicemia and their Index Mothers Admitted at ARTH (n = 392).

Variables	Category	Culture status		Total n (%)
		Positive	Negative	
Age in days	≤ 7	202 (63.3)	117 (36.7)	319 (81.4)
	> 7	44 (60.3)	29 (39.7)	73 (18.6)
Sex of neonate	Male	158 (62.0)	97 (38.0)	255 (65.1)
	Female	88 (64.2)	49 (35.8)	137 (34.9)
Gestational age	Preterm	125 (72.3)	48 (27.7)	173 (44.1)
	Term	121 (55.3)	98 (44.7)	219 (55.9)
Birth weight	LBW	111 (68.1)	52 (31.9)	163 (41.6)
	NBW	135 (59.0)	94 (41.0)	229 (58.4)
PROM(>18hrs)	Yes	39 (52.7)	35 (47.3)	74 (18.9)
	No	207 (65.1)	111 (34.9)	318 (81.1)
Resuscitated at Birth	Yes	16 (45.7)	19 (54.3)	35 (8.9)
	No	230 (64.4)	127 (35.6)	357 (91.1)
Duration of labor	Normal	232 (62.5)	139 (37.5)	371 (94.6)
	Prolonged	14 (66.7)	7 (33.3)	21 (5.4)
Multiple births	Yes	43 (72.9)	16 (27.1)	59 (15.0)
	No	203 (61.0)	130 (39.0)	333 (85.0)
Mode of delivery	SVD	166 (63.4)	96 (36.6)	262 (66.9)
	ID	7 (53.8)	6 (46.2)	13 (3.3)
	CS	73 (62.4)	44 (37.6)	117 (29.9)
Place of delivery	Home	17 (70.8)	7 (29.2)	24 (6.1)
	Health center	72 (66.7)	36 (33.3)	108 (27.6)
	Hospital	157 (60.4)	103 (39.6)	260 (66.3)
Admission RR	< 60	37 (37.8)	61 (62.2)	98 (25.0)
	≥ 60	209 (71.1)	85 (28.9)	294 (75.0)
Admission temp. (⁰C)	< 36.5	85 (76.6)	26 (23.4)	111 (28.3)
	36.5–37.5	106 (56.4)	82 (43.6)	188 (48.0)
	>37.5	55 (59.1)	38 (40.9)	93 (23.7)
Sepsis category	EOS	179(63.5)	103(36.5)	282 (71.9)
	LOS	67(60.9)	43(39.1)	110 (28.1)
Length of hospital stay	< 7	103 (53.6)	89 (46.4)	192 (49.0)
	≥ 7	143 (71.5)	57 (28.5)	200 (51.0)
5-minute Apgar score	<7	62(83.8)	12(16.2)	74 (18.9)
	≥7	184(57.9)	134(42.1)	318(81.1)
ANC follow-up	Yes	241 (62.6)	144 (37.4)	385 (98.4)
	No	5 (71.4)	2 (28.6)	7 (1.6)
Intrapartum fever	Yes	18 (48.6)	19 (51.4)	37 (9.5)
	No	228 (64.2)	127 (35.8)	355 (90.5)
Chorioamnionitis	Yes	79 (80.6)	19 (19.4)	98 (25.0)
	No	167 (56.8)	127 (43.2)	294 (75.0)
Maternal UTI/STI During Pregnancy	Yes	18 (78.3)	5 (21.7)	23 (5.9)
	No	228 (61.8)	141 (38.2)	369 (94.1)
Antibiotic treatment of the mother	Yes	59 (59.0)	41 (41.0)	100 (25.5)
	No	187 (64.0)	105 (36.0)	292 (74.5)
WBC Count (/mm³)	<5000	10(55.6)	8 (44.4)	18 (4.6)
	5000–20000	211 (64.5)	116 (35.5)	327 (83.4)
	>20000	25 (53.2)	22 (46.8)	47 (12.0)
CRP	<1 mg/dL	78 (62.9)	46 (37.1)	124 (31.5)
	≥1 mg/dL	168 (62.7)	100 (37.3)	268 (68.5)

***LBW:*** Low Birth Weight, ***CS:*** Cesarean Section, ***SVD:*** Spontaneous Vaginal Delivery, ***APGAR:*** Activity, Pulse, Grimace, Appearance, and Respiration, ***CRP:*** C-Reactive Protein; ***EBF*** = Exclusive Breast Feeding, ***ID:*** Instrumental Delivery; ***UTI:*** Urinary Tract Infections, ***STI*** = Sexually Transmitted Infection, ***ANC:*** Antenatal Care, ***PROM:*** Prolonged Rupture of Membrane, ***APGAR:*** Activity, Pulse, Grimace, Appearance, and Respiration. ***RR:*** Respiratory Rate.

### Prevalence of culture-confirmed sepsis

**Fig 1:**
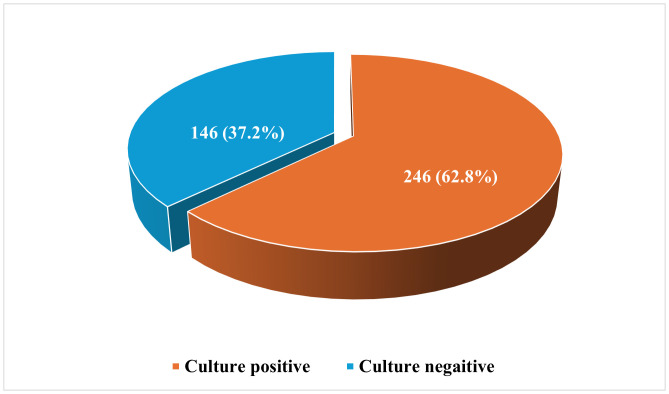
Prevalence of culture-confirmed sepsis among neonates admitted at ARTH (n =  392).

A total of 392 neonates with clinically suspected sepsis were included in the analysis. Of these, 246 had positive blood cultures, yielding a prevalence of culture-confirmed neonatal sepsis of 62.8% (95% CI: 57.9–67.1) (Fig 1). Among neonates with culture-confirmed sepsis (n = 246), 121 (49.2%) were born at term and 125 (50.8%) were preterm.

### Bacteriological profile of neonatal septicemia

**Fig 2:**
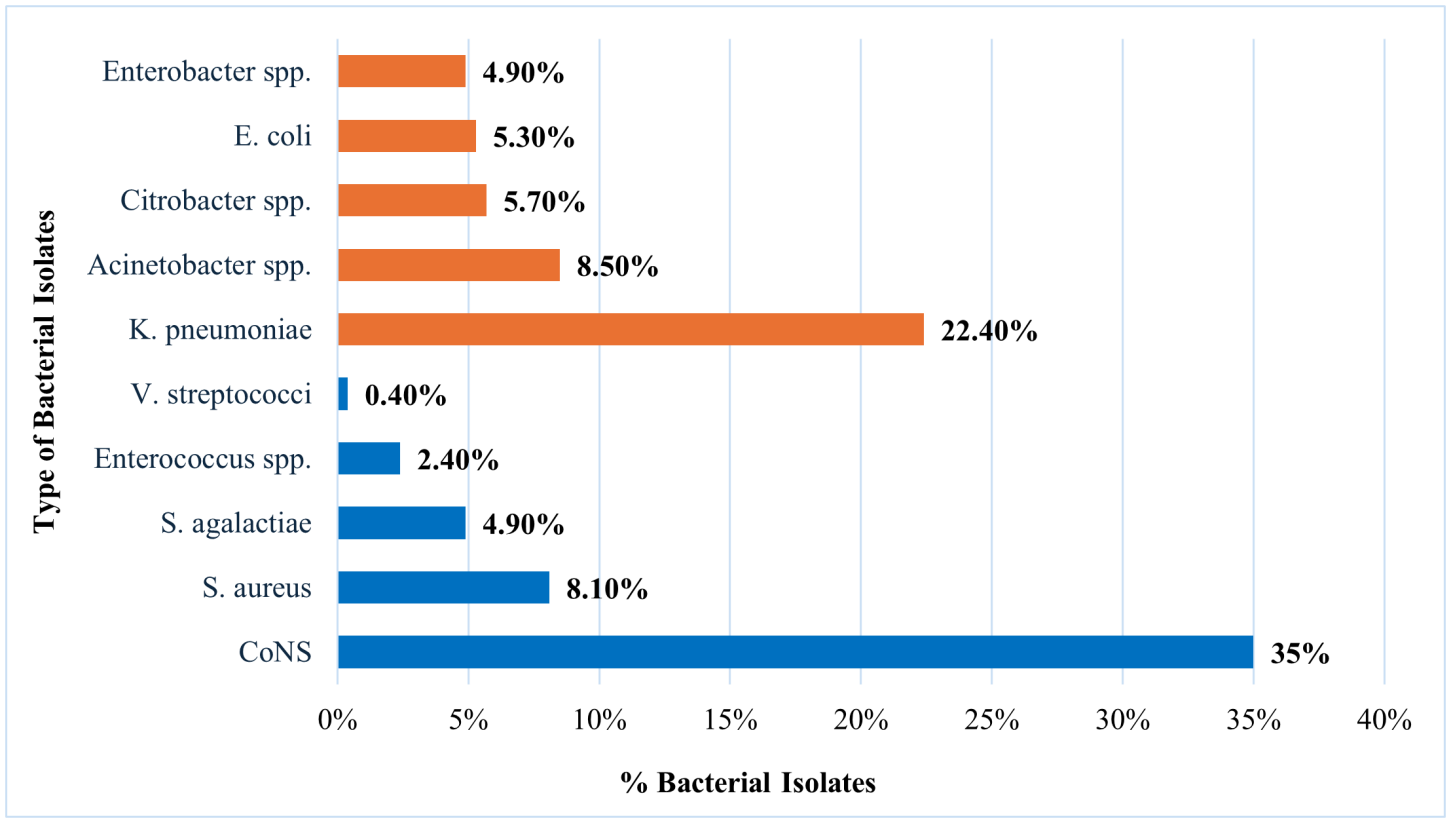
Distribution of commonly isolated bacteria from blood cultures among neonates with confirmed septicemia at ARTH.

A total of 246 bacterial isolates were recovered from neonates with culture-confirmed sepsis. Gram-positive organisms accounted for 125/246 isolates (50.8%), and Gram-negative organisms for 121/246 (49.2%). Among Gram-positive isolates, *CoNS* predominated (86/246, 35.0%; 86/125, 68.8%). Other Gram-positive pathogens included *S. aureus* (20/246, 8.1%), *Streptococcus agalactiae* (12/246, 4.9%), and *Enterococcus* spp. (6/246, 2.4%). Among Gram-negative isolates, *K. pneumoniae* was most frequently identified (55/246, 22.4%; 55/121, 45.5%), followed by *Acinetobacter* spp. (21/246, 8.5%), *Citrobacter* spp. (14/246, 5.7%), *E. coli* (13/246, 5.3%), and *Enterobacter* spp. (12/246, 4.9%) (Table 2). Overall, *CoNS* and *K. pneumoniae* were the leading pathogens (Fig 2).

**Table 2:** Bacteriological Profile of Neonates with Culture-Confirmed Sepsis across Early-Onset and Late-Onset Sepsis at the Neonatal Intensive Care Unit of Admitted at ARTH.

Bacterial Isolates	EONS N (%)	LONS N (%)	Total isolates N (%)
**Gram-positive organisms**	**88 (70.4)**	**37 (29.6)**	**125 (50.8)**
CoNS	57 (66.3)	29 (33.7)	86 (35.0)
Staphylococcus aureus	17 (85.0)	3 (15.0)	20 (8.1)
Streptococcus agalactiae	10 (83.3)	2 (16.7)	12 (4.9)
Enterococcus spp.	4 (66.7)	2 (33.3)	6 (2.4)
Viridians streptococci	0 (0.0)	1 (100.0)	1 (0.4)
**Gram-negative organisms**	**91(75.2)**	**30(24.8)**	**121(49.2)**
Klebsiella pneumoniae	41 (74.5)	14 (25.5)	55 (22.4)
Acinetobacter spp.	16 (76.2)	5 (23.8)	21 (8.5)
Citrobacter spp.	13 (92.9)	1 (7.1)	14 (5.7)
Escherichia coli	9 (69.2)	4 (30.8)	13 (5.3)
Enterobacter aerogenes	8 (66.7)	4 (33.3)	12 (4.9)
Pseudomonas aeruginosa	3 (60.0)	2 (40.0)	5 (2.0)
Serratia species	1 (100.0)	0 (0.0)	1 (0.4)
**Total**	**179 (72.8)**	**67 (27.2)**	**246 (100)**

**Key: N:** number of isolates; **CoNS:** coagulase-negative Staphylococcus; **EONS:** early-onset neonatal sepsis; **LONS:** late-onset neonatal sepsis; **spp.**: species.

Early-onset neonatal sepsis (EONS) accounted for 179/246 cases (72.8%), and late-onset neonatal sepsis (LONS) for 67/246 (27.2%). Gram-negative organisms were more frequently isolated in EONS (91/121, 75.2%), whereas 37/125 (29.6%) Gram-positive isolates occurred in LONS. Species-level analysis showed that *S. aureus* (17/20, 85.0%), *S. agalactiae* (10/12, 83.3%), and *Enterococcus* spp. (4/6, 66.7%) were predominantly associated with EONS. *CoNS* constituted the majority of Gram-positive isolates in LONS. Among Gram-negative organisms, *Citrobacter* spp. (13/14, 92.9%), *Acinetobacter* spp. (16/21, 76.2%), and *K. pneumoniae* (41/55, 74.5%) were primarily recovered from EONS cases (Table 2).

### Antimicrobial susceptibility pattern of bacterial isolates from neonatal septicemia

#### Gram-positive bacteria.

Among the 125 Gram-positive isolates, the overall resistance rate across tested antimicrobial agents was 53.6%. Vancomycin exhibited the highest susceptibility (91.9%), followed by trimethoprim–sulfamethoxazole (76.3%), amikacin (72.9%), and clindamycin (69.8%). High resistance rates were observed for ampicillin (91.9%) and ciprofloxacin (80.5%). Substantial resistance was also noted against cephalosporins, including ceftazidime (83.3%), cefoxitin (66.1%), ceftriaxone (65.3%), and cefotaxime (48.5%). Species-specific analysis revealed variable resistance patterns: CoNS (n = 86): 95.3% resistant to ampicillin (82/86) and 75.6% to cefoxitin (65/86), indicating a high prevalence of methicillin resistance. S. aureus (n = 20): 95.0% resistant to ampicillin (19/20) and 85.0% to amikacin (17/20), but largely susceptible to trimethoprim–sulfamethoxazole (16/20, 80% susceptible) and vancomycin (18/20, 90% susceptible). Streptococcus agalactiae (n = 12): Moderate-to-high resistance was observed to ampicillin (7/12, 58.3%) and gentamicin (8/12, 66.7%). Enterococcus spp. (n = 6): Uniform resistance to ampicillin, gentamicin, and ceftriaxone (6/6, 100%), while all isolates remained susceptible to vancomycin (6/6, 100%) (Table 3).

**Table 3:** Antimicrobial susceptibility patterns of Gram-positive bacterial isolates from neonatal sepsis cases at ARTH.

Antimicrobial Agent	CoNS n = 86		S. aureus n = 20		S. agalactiae n = 12		Enterococcus spp. n = 6		TOTAL n = 124	
	S (%)	R (%)	S (%)	R (%)	S (%)	R (%)	S (%)	R (%)	S (%)	R (%)
Ampicillin	4 (4.7)	82 (95.3)	1 (5.0)	19 (95.0)	5 (41.7)	7 (58.3)	0 (0.0)	6 (100)	10 (8.1)	114 (91.9)
Ciprofloxacin	13 (15.1)	73 (84.9)	2 (10.0)	18 (90.0)	8 (66.7)	4 (33.3)	NT	NT	23 (19.5)	95 (80.5)
Cotrimoxazole	NT	NT	16 (80.0)	4 (20.0)	8 (66.7)	4 (33.3)	5 (83.3)	1 (16.7)	29 (76.3)	9 (23.7)
Amoxicillin-Clav	44 (51.2)	42 (48.8)	13 (65.0)	7 (35.0)	7 (58.3)	5 (41.7)	4 (66.7)	2 (33.3)	68 (54.8)	56 (45.2)
Gentamicin	NT	NT	8 (40.0)	12 (60.0)	4 (33.3)	8 (66.7)	0 (0.0)	6 (100)	12 (31.6)	26 (68.4)
Erythromycin	NT	NT	5 (25.0)	15 (75.0)	5 (41.7)	7 (58.3)	NT	NT	10 (31.2)	22 (68.8)
Chloramphenicol	27 (31.4)	59 (68.6)	6 (30.0)	14 (70.0)	5 (41.7)	7 (58.3)	4 (66.7)	2 (33.3)	42 (33.9)	82 (66.1)
Amikacin	74 (86.0)	12 (14.0)	3 (15.0)	17 (85.0)	9 (75.0)	3 (25.0)	NT	NT	86 (72.9)	32 (27.1)
Clindamycin	68 (79.1)	18 (20.9)	6 (30.0)	14 (70.0)	NT	NT	NT	NT	74 (69.8)	32 (30.2)
Cefoxitin	21 (24.4)	65 (75.6)	11 (55.0)	9 (45.0)	4 (33.3)	8 (66.7)	6 (100)	0 (0.0)	42 (33.9)	82 (66.1)
Ceftriaxone	30 (34.9)	56 (65.1)	8 (40.0)	12 (60.0)	5 (41.7)	7 (58.3)	0 (0.0)	6 (100)	43 (34.7)	81 (65.3)
Cefotaxime	49 (57.0)	37 (43.0)	11 (55.0)	9 (45.0)	6 (50.0)	6 (50.0)	1 (16.7)	5 (83.3)	67 (51.5)	63 (48.5)
Ceftazidime	NT	NT	NT	NT	2 (16.7)	10 (83.3)	NT	NT	2 (16.7)	10 (83.3)
Cefepime	39 (45.3)	47 (54.7)	3 (15.0)	17 (85.0)	9 (75.0)	3 (25.0)	NT	NT	68 (49.3)	70 (50.7)
Vancomycin	79 (91.9)	7 (8.1)	18 (90.0)	2 (10.0)	10 (83.3)	2 (16.7)	6 (100)	0 (0.0)	113 (91.9)	10 (8.1)
Meropenem	NT	NT	NT	NT	NT	NT	NT	NT	–	–
Imipenem	NT	NT	NT	NT	NT	NT	NT	NT	–	–

#### Gram-negative bacteria.

Among Gram-negative isolates (n = 121), 59.9% demonstrated resistance to the tested antimicrobials. Overall, amikacin demonstrated the greatest activity (85.8%), followed by carbapenems (75.8%), while cefepime showed moderate effectiveness (64.2%). Notably, resistance was extremely high to ampicillin (92.5%) and third-generation cephalosporins, including ceftriaxone (90%), ceftazidime (93.3%), and cefotaxime (65.8%). Elevated resistance rates were also observed for ciprofloxacin (71.7%), gentamicin (72.5%), and trimethoprim–sulfamethoxazole (84.2%) (Table 4). Species-specific patterns demonstrated notable variability: Klebsiella pneumoniae retained high susceptibility to amikacin (96.4%) and imipenem (92.7%) but exhibited complete resistance to ampicillin and cotrimoxazole. Acinetobacter spp. showed extensive resistance, remaining largely susceptible only to imipenem (95.2%). Citrobacter spp., *Escherichia coli*, and Enterobacter spp. were fully susceptible to amikacin, with preserved susceptibility to carbapenems or cefepime, but resistant to most β-lactams. Pseudomonas aeruginosa remained fully susceptible to amikacin and cefotaxime while resistant to ampicillin and cotrimoxazole (Table 4).

**Table 4:** Antimicrobial susceptibility patterns of Gram-negative bacterial isolates from neonates with clinically diagnosed sepsis admitted to ARTH.

Antimicrobial Agent	K. pneumoniae n = 55		Acinetobacter spp. n = 21		Citrobacter spp. n = 14		E. coli n = 13		Enterobacter spp. n = 12		P. aeruginosa n = 5		TOTAL n = 120	
	S (%)	R (%)	S (%)	R (%)	S (%)	R (%)	S (%)	R (%)	S (%)	R (%)	S (%)	R (%)	S (%)	R (%)
**Ampicillin**	0 (0)	55 (100)	0 (0)	21 (100)	0 (0)	14 (100)	9 (69.2)	4 (30.8)	0 (0)	12 (100)	0 (0)	5 (100)	9 (7.5)	111 (92.5)
**Ciprofloxacin**	14 (25.5)	41 (74.5)	7 (33.3)	14 (66.7)	3 (21.4)	11 (78.6)	4 (30.8)	9 (69.2)	2 (16.7)	10 (83.3)	4 (80)	1 (20)	34 (28.3)	86 (71.7)
**Cotrimoxazole**	0 (0)	55 (100)	8 (38.1)	13 (61.9)	0 (0)	14 (100)	5 (38.5)	8 (61.5)	6 (50)	6 (50)	0 (0)	5 (100)	19 (15.8)	101 (84.2)
**Gentamicin**	5 (9.1)	50 (90.9)	6 (28.6)	15 (71.4)	9 (64.3)	5 (35.7)	3 (23.1)	10 (76.9)	8 (66.7)	4 (33.3)	2 (40)	3 (60)	33 (27.5)	87 (72.5)
**Chloramphenicol**	29 (52.7)	26 (47.3)	14 (66.7)	7 (33.3)	0 (0)	14 (100)	6 (46.2)	7 (53.8)	9 (75)	3 (25)	1 (20)	4 (80)	59 (49.2)	61 (50.8)
**Amikacin**	53 (96.4)	2 (3.6)	6 (28.6)	15 (71.4)	14 (100)	0 (0)	13 (100)	0 (0)	12 (100)	0 (0)	5 (100)	0 (0)	103 (85.8)	17 (14.2)
**Ceftriaxone**	1 (1.8)	54 (98.2)	3 (14.3)	18 (85.7)	0 (0)	14 (100)	5 (38.5)	8 (61.5)	2 (16.7)	10 (83.3)	1 (20)	4 (80)	10 (9.0)	108 (90.0)
**Cefotaxime**	13 (23.6)	42 (76.4)	16 (76.2)	5 (23.8)	0 (0)	14 (100)	4 (30.8)	9 (69.2)	3 (25)	9 (75)	5 (100)	0 (0)	41 (34.2)	79 (65.8)
**Ceftazidime**	3 (5.6)	53 (94.6)	1 (4.8)	20 (95.2)	0 (0)	14 (100)	2 (15.4)	11 (84.6)	1 (8.3)	11 (91.7)	2 (40)	3 (60)	6 (7.7)	112 (93.3)
**Cefepime**	43 (78.2)	12 (21.8)	15 (71.4)	6 (28.6)	0 (0)	14 (100)	3 (23.1)	10 (76.9)	12 (100)	0 (0)	4 (80)	1 (20)	77 (64.2)	43 (35.8)
**Meropenem**	50 (90.9)	5 (9.1)	13 (61.9)	8 (38.1)	14 (100)	0 (0)	13 (100)	0 (0)	10 (83.3)	2 (16.7)	4 (80)	1 (20)	91 (75.8)	29 (24.2)
**Imipenem**	51 (92.7)	4 (7.3)	20 (95.2)	1 (4.8)	0 (0)	14 (100)	8 (61.5)	5 (38.5)	11 (91.7)	1 (8.3)	1 (20)	4 (80)	91 (75.8)	29 (24.2)

**Key:** S: sensitive; R: resistant; CONS: coagulase-negative staphylococci; NT: not tested.

### Multidrug-resistance pattern of bacterial isolates

Multidrug resistance (MDR), defined as resistance to ≥3 antimicrobial classes, was more common in Gram-negative bacteria (97/121, 80.2%) than Gram-positive bacteria (72/125, 57.6%). Among Gram-positive isolates, Enterococcus spp. had the highest MDR rate (5/6, 83.3%), followed by CoNS (56/86, 65.1%) and *S. aureus* (11/20, 55.0%), while no MDR was detected in *S. agalactiae*. In Gram-negative bacteria, Acinetobacter spp. exhibited the highest MDR prevalence (20/21, 95.2%), followed by *K. pneumoniae* (49/55, 89.1%). High MDR rates were also observed in *P. aeruginosa* (4/5, 80.0%) and *E. coli* (9/13, 69.2%), whereas Enterobacter spp. showed the lowest proportion of MDR isolates within this group (5/12, 41.7%) (Table 5).

**Table 5:** Patterns of multidrug resistance among bacterial isolates from neonates with suspected septicemia at ARTH.

Bacterial isolates (n)	Degree of microbial resistance (R0 - ≥ R4)					Total MDR Isolates ≥ R3 n (%)
	R0 N (%)	R1 N (%)	R2 N (%)	R3 N (%)	≥R4 N (%)	
**Gram positive (n = 125)**	**15 (12.0)**	**19 (15.2)**	**18 (14.4)**	**41 (32.8)**	**31 (24.8)**	**72 (57.6)**
CoNS (n = 86, 68.8%)	7 (8.1)	12 (14.0)	11 (12.8)	34 (39.5)	22 (25.6)	56 (65.1)
*S. aureus* (n = 20, 16.0%)	2 (10.0)	3 (15.0)	4 (20.0)	6 (30.0)	5 (25.0)	11 (55.0)
*S. agalactiae* (n = 12, 9.6%)	6 (50.0)	4 (33.3)	2 (16.7)	0 (0.0)	0 (0.0)	0 (0.0)
*Enterococcus spp.* (n = 6, 4.8%)	0 (0.0)	0 (0.0)	1 (16.7)	1 (16.7)	4 (66.7)	5 (83.3)
*S. viridians* (n = 1, 0.8%)	0 (0.0)	1 (100.0)	0 (0.0)	0 (0.0)	0 (0.0)	0 (0.0)
**Gram negative (n = 121)**	**2 (1.7)**	**6 (5.0)**	**15 (12.4)**	**45 (37.2)**	**51 (42.1)**	**97 (80.2)**
*K. pneumoniae* (n = 55, 45.45%)	0 (0.0)	0 (0.0)	6 (10.9)	23 (41.8)	26 (47.3)	49 (89.1)
*Acinetobacter spp.* (n = 21, 17.36%)	1 (4.8)	0 (0.0)	1 (4.8)	7 (33.3)	13 (61.9)	20 (95.2)
*Citrobacter spp.* (n = 14, 11.57%)	1 (7.1)	2 (14.3)	2 (14.3)	5 (35.7)	4 (28.6)	9 (64.3)
*E. coli* (n = 13, 10.74%)	0 (0.0)	1 (7.7)	3 (23.1)	6 (46.2)	3 (23.1)	9 (69.2)
*Enterobacter spp.* (n = 12, 9.92%)	0 (0.0)	4 (33.3)	3 (25.0)	3 (25.0)	2 (16.7)	5 (41.7)
*P. aeruginosa* (n = 5, 4.13%)	0 (0.0)	0 (0.0)	1 (20.0)	1 (20.0)	3 (60.0)	4 (80.0)
*Serratia spp.* (n = 1, 0.83%)	0 (0.0)	0 (0.0)	0 (0.0)	0 (0.0)	1 (100.0)	1 (100.0)

**Key:** CoNS = coagulase-negative Staphylococcus; spp.: species; R0: susceptible to all antibiotics; **R1**: resistance to one category of antimicrobial agent; R2: resistance to two different categories of antimicrobial agents; R3: resistance to three different categories of antimicrobial agents; R4: ≥ resistance to four different categories of antimicrobial agents; MDR: multidrug resistance.

### Predictors of culture-confirmed neonatal sepsis

Multivariable logistic regression analysis was conducted to identify factors independently associated with culture-confirmed neonatal sepsis, controlling for potential confounders. Variables with p-values <0.25 in bivariate analysis were included in the multivariable model. The analysis demonstrated that both neonatal and maternal/clinical factors significantly influenced the likelihood of culture-confirmed infection. Preterm birth was associated with more than a twofold increase in the odds of culture-proven sepsis compared to term neonates (AOR: 2.20; 95% CI: 1.07–4.98; p = 0.048). Neonates presenting with tachypnea at admission exhibited an approximately fivefold higher risk of culture-confirmed sepsis relative to those with normal respiratory rates (AOR: 4.79; 95% CI: 2.73–8.41; p < 0.001). Similarly, hypothermia at admission (temperature <36.5°C) was associated with a twofold increased likelihood of positive blood cultures compared with normothermic neonates (AOR: 2.35; 95% CI: 1.12–4.92; p = 0.010). Prolonged hospital stay was also a significant predictor, with affected neonates demonstrating 2.5 times higher odds of developing culture-confirmed sepsis than those hospitalized for shorter durations (AOR: 2.51; 95% CI: 1.52–4.16; p < 0.001). Maternal chorioamnionitis emerged as a strong risk factor, with neonates born to affected mothers exhibiting nearly fourfold higher odds of culture-confirmed sepsis (AOR: 4.49; 95% CI: 2.35–8.80; p < 0.001). Finally, a five-minute Apgar score below seven was independently associated with an almost fourfold increase in the risk of positive blood culture compared with neonates who had normal Apgar scores (AOR: 3.95; 95% CI: 1.87–8.34; p < 0.001) (Table 6).

**Table 6:** Bivariable and Multivariable Logistic Regression Analysis of Factors Associated with Culture-Confirmed Neonatal Sepsis Among Newborns Admitted at ARTH (n = 392).

Variables	Category	Blood culture result		COR (95% CI)	AOR (95% CI)	P-value
		Positive n (%)	Negative n (%)			
Gestational age	Preterm	126(72.8)	47(27.2)	2.21 (1.44-3.39)	2.20(1.07-4.98)	0.048*
	Term	120(54.8)	99(45.2)	1	1	
Birth weight	LBW	112(68.7)	51(31.3)	1.56(1.02-2.38)	0.72(0.32-1.65)	0.44
	NBW	134(58.5)	95(41.5)	1	1	
PROM(>18hrs)	Yes	39(52.7)	35(47.3)	0.60(0.36-0.99)	0.57(0.31-1.05)	0.072
	No	207(65.1)	111(34.9)	1	1	
Resuscitated at Birth	Yes	16(45.7)	19(54.3)	0.47(.23−.94)	0.45(0.20-1.01)	0.054
	No	230(64.4)	127(35.6)	1	1	
Admission RR	< 60	37(37.8)	61(62.2)	1	1	< 0.001**
	≥ 60	209(71.1)	85(28.9)	4.05(2.51-6.55)	4.79(2.73-8.41)	
Admission Temp. (⁰C)	< 36.5	85(76.6)	26(23.4)	2.36(1.29-4.31)	2.35(1.12-4.97)	0.010*
	36.5–37.5	107(56.9)	81(43.1)	0.95(0.58-1.58)	0.92(0.50-1.69)	
	>37.5	54(58.1)	39(41.9)	1	1	
Sepsis Category	EOS	179(63.5)	103(36.5)	1	1	0.801
	LOS	67(60.9)	43(39.1)	0.89(0.57-1.41)	0.93(0.54-1.60)	
Length of Hospital Stay	< 7 days	104(54.2)	88(45.8)	1	1	< 0.001**
	≥ 7 days	142(71)	58(29)	2.07(1.37-3.14)	2.51(1.52-4.16)	
Chorioamnionitis	Yes	79(80.6)	19(19.4)	3.16(1.82-5.49)	4.49(2.35-8.60)	< 0.001**
	No	167(56.8)	127(43.2)	1	1	
5^th^ min Apgar score	<7	62(83.8)	12(16.2)	3.76(1.95-7.26)	3.95(1.87-8.34)	< 0.001**
	≥7	184(57.9)	134(42.1)	1	1	
Multiple birth	Yes	43(72.9)	16(27.1)	1.72(0.93-3.18)	1.65(0.86-3.35)	0.164
	No	203(61)	130(39)	1	1	

*Key:* AOR: Adjusted Odds Ratio; COR: crude odds ratio; CI: Confidence Interval; * to show p-value < 0.05; ** to show p value < 0.01; 1: Reference; *APGAR:* Activity, Pulse, Grimace, Appearance, and Respiration; EOS: early-onset of sepsis; *LOS*: late-onset of sepsis; *NBW*: normal birth weight; Temp: temperature on admission; *LBW*: Low Birth Weight; *PROM*: Prolonged Rupture of Membrane.

## Discussion

Neonatal sepsis remains a major cause of morbidity and mortality during the first month of life, particularly in low- and middle-income countries, where timely diagnosis and appropriate antibiotic therapy are critical. The increasing prevalence of antimicrobial resistance further complicates empiric treatment strategies, making knowledge of local pathogen profiles and susceptibility patterns essential for guiding effective therapy [36]. At ARTH, limited local data on pathogens and resistance patterns hampers efforts to reduce neonatal morbidity and mortality. This study provides essential insights into the bacteriological profile, antimicrobial susceptibility patterns, and predictors of neonatal sepsis in a tertiary care setting in Southeast Ethiopia, while evaluating the concordance between WHO-recommended antibiotic regimens and local resistance patterns.

The overall culture-confirmed sepsis rate in our study was 62.8% (246/392), considerably higher than previous reports from Ethiopian hospitals, such as St. Paul’s Hospital Millennium Medical College (21%) [37], University of Gondar Hospital (25.4%) [34], Dessie Comprehensive Hospital (27.2%) [26], Asella Teaching and Referral Hospital (29.4%) [23], Hawassa University Hospital (36.5%) [38], Ayder Specialized Hospital (36.6%) [24], and Felege-Hiwot Referral Hospital (41.3%) [39]. This variability may be attributed to differences in blood culture techniques, laboratory capacity, infection prevention practices, referral patterns, study design, and neonatal infection epidemiology. The high rate in our study likely reflects ARTH’s role as a major referral hospital, receiving a high proportion of complicated neonatal and maternal cases. Globally, our culture positivity rate aligns with reports from Pakistan (66.4%) [40] and Uganda (59%) [35], but exceeds rates in India (39.4%) [41], Nepal (10.8%) [14], Bhutan (14%) [42], Ghana (21%) [13], and Palestine (14.6%) [43]. Conversely, it is lower than in Zambia (69.8%) [44], Northern Ghana (70.3%) [33], and Tanzania (72%) [45]. Such variability reflects regional differences in hygiene practices, antimicrobial use, laboratory protocols, study designs, and infection control practices. These findings underscore the persistent burden of neonatal sepsis in low-resource settings and the urgent need for improved prevention, diagnosis, and management strategies.

Early-onset neonatal sepsis (EONS) accounted for a higher proportion of positive cultures than late-onset sepsis (LONS) (72.8% vs. 27.2%), consistent with studies from Ethiopia [37] and Uganda (67.4%) [46]. The predominance of EONS likely reflects vertical transmission from the maternal genital tract and early horizontal transmission in NICUs or delivery rooms, particularly after unhygienic obstetric practices [47]. In contrast, LONS usually arises postnatally from environmental exposure, and its lower prevalence may reflect improved hygiene practices. Findings from India, however, reported LONS as the predominant form (68.6%) [41], potentially influenced by obstetric antibiotic use affecting neonatal blood culture results through transplacental transfer [48].

In terms of bacterial distribution, Gram-positive (50.8%) and Gram-negative (49.2%) isolates were nearly equal, aligning with a study from western Nepal [49]. This differs from other Ethiopian and international reports, where Gram-negative bacteria, particularly Klebsiella pneumoniae, Acinetobacter spp., and Escherichia coli, predominate [14,34,35,38,39,42]. Variations may reflect differences in population characteristics, healthcare settings, antimicrobial use, infection-control practices, and regional microbial ecology. Temporal shifts in pathogen prevalence and laboratory diagnostic capacities may also contribute. Among Gram-positive isolates, coagulase-negative Staphylococci (CoNS) and *Staphylococcus aureus* were predominant, consistent with findings from Nigeria [50] and Ghana [51]. CoNS infections are commonly nosocomial, arising from healthcare workers and the hospital environment, and may colonize the umbilical stump, nose, and groin early in life [52,53]. In this study, 35% of CoNS isolates likely represented contamination; however, clinically significant infections remain concerning, with 75.6% showing cefoxitin resistance yet retaining vancomycin susceptibility, highlighting emerging multidrug-resistant CoNS in ARTH NICUs.

Among Gram-negative bacteria, K. pneumoniae was most frequent, followed by Acinetobacter spp. and Citrobacter, consistent with previous studies [32,42,54,55]. Klebsiella’s high prevalence likely reflects patient-to-patient spread via contaminated hands, environment, or medical devices such as ventilators and catheters. These bacteria are ubiquitous, capable of surviving in hospital settings and infecting immunocompromised patients [56]. Supporting this, Bitew et al. isolated Klebsiella strains from medical devices and NICU environments in Hawassa Hospital [57]. Acinetobacter spp. and Citrobacter, associated with early-onset sepsis, reflect nosocomial transmission and pose therapeutic challenges due to multidrug resistance [58]. These pathogens’ persistence in hospital environments underscores the importance of stringent infection-control practices.

Antimicrobial susceptibility testing demonstrated substantial resistance to commonly used empirical antibiotics. Among Gram-positive isolates, vancomycin showed the highest efficacy (91.9%), followed by trimethoprim–sulfamethoxazole (76.3%), amikacin (72.9%), and clindamycin (69.8%). In contrast, resistance was particularly high against ampicillin (91.9%) and multiple cephalosporins. Similarly, Gram-negative isolates exhibited the greatest susceptibility to amikacin (85.8%), carbapenems (meropenem and imipenem, 75.8%), and cefepime (64.2%), while demonstrating marked resistance to ampicillin (92.5%) and third-generation cephalosporins (90–93.3%). These findings are consistent with previous Ethiopian reports, including approximately 89% resistance of *Klebsiella spp.* to ceftriaxone and cefepime in Addis Ababa NICUs [59], and similar trends in India [55], Tanzania [60], and Pakistan [61]. Although ampicillin and gentamicin remain widely recommended as first-line therapy for neonatal sepsis [62–64], our findings reveal substantial resistance to ampicillin and third-generation cephalosporins across both Gram-positive and Gram-negative isolates. This pattern reflects the declining efficacy of beta-lactam antibiotics, largely attributable to widespread beta-lactamase production [21]. In contrast, preserved susceptibility to amikacin and carbapenems supports their continued utility in settings with high prevalence of extended-spectrum beta-lactamase (ESBL) and multidrug-resistant (MDR) organisms, consistent with WHO guidance [22].

The overall MDR prevalence (68.7%), notably higher among Gram-negatives (80.2%) than Gram-positives (57.6%), is alarming. MDR rates were highest in *Acinetobacter spp.* (95.2%), *K. pneumoniae* (89.1%), and *Enterococcus spp.* (83.3%), reflecting their role as biofilm-forming, environmentally resilient nosocomial pathogens. This aligns with a Jimma study reporting 88.4% MDR among isolates, including 100% of Klebsiella and high rates in Acinetobacter and CoNS [65], national pooled estimates (~80.5%) [66], and other Ethiopian studies documenting high ampicillin resistance and ESBL production [67]. These data underscore an urgent need for strengthened antimicrobial stewardship and continuous resistance surveillance to safeguard effective neonatal sepsis management [68]. Our findings substantiate concerns that first- and second-line empiric therapies may be losing effectiveness in many Ethiopian tertiary hospitals.

Several neonatal and maternal factors were significantly associated with culture-confirmed sepsis. Preterm birth (<37 weeks) doubled the odds of culture-proven sepsis, consistent with studies in Ethiopia [25,37,39,69], and global studies [32,70,71]. Increased vulnerability likely stems from reduced neutrophil production, lower immunoglobulin levels, absent transplacental IgA transfer, and immature mucosal defenses [72,73]. Neonates presenting with tachypnea had nearly fivefold higher odds of culture-confirmed sepsis, reflecting systemic inflammation or metabolic disturbances, aligning with prior Ethiopian studies [34,39]. Hypothermia at admission also doubled the odds of positive blood cultures, highlighting the limited thermoregulatory capacity of neonates and its role as an early sepsis sign [34]. These associations likely reflect the nonspecific but physiologically significant responses of neonates to infection. Maternal chorioamnionitis increased sepsis risk fourfold, likely due to ascending infection, preterm birth, and longer hospitalization, emphasizing the need for maternal health monitoring and intrapartum antibiotic prophylaxis [74]. A low fifth-minute Apgar score (<7) nearly quadrupled the risk, consistent with studies in Mekelle and Indonesia [75,76]. While the first-minute Apgar score reflects cord blood pH and intrapartum distress, the five-minute score better reflects neonatal adaptation post-resuscitation and vulnerability to infection [77]. Lastly, hospitalization exceeding seven days was associated with a 2.5-fold increased risk of sepsis, in line with findings from Mekelle [24], likely reflecting greater exposure to nosocomial pathogens and underscoring the need for stringent infection control measures [78]. Collectively, these factors underscore the need for vigilant monitoring and early intervention in high-risk neonates.

### Limitations of the study

This study’s single-center design limits its generalizability. The absence of molecular typing and advanced resistance characterization (e.g., MRSA, ESBL, VRE, and MDR *Pseudomonas aeruginosa*) restricted detailed analysis of resistance mechanisms and transmission dynamics. In addition, reliance on retrospective chart review may have resulted in incomplete clinical data, and the study could not definitively distinguish maternal from nosocomial sources of infection, which restricts interpretation of transmission dynamics. Multicenter studies incorporating advanced microbiological methods are needed to validate and expand upon these findings.

## Conclusion and recommendation

This study revealed a high prevalence of culture-confirmed neonatal sepsis, with a substantial burden of multidrug-resistant pathogens. High resistance rates were observed to commonly used first-line antibiotics, particularly ampicillin and third-generation cephalosporins, while the prevalence of multidrug-resistant Gram-positive isolates remained largely susceptible to vancomycin, trimethoprim–sulfamethoxazole, amikacin, and clindamycin, whereas Gram-negative isolates were most sensitive to amikacin, meropenem, and cefepime, highlighting amikacin and carbapenems as the most reliable empiric treatment options. Independent predictors of neonatal bacteremia included preterm birth, elevated respiratory rate at admission, hypothermia, prolonged hospitalization, maternal chorioamnionitis, and low fifth-minute Apgar scores.

These findings underscore the urgent need for routine local surveillance of pathogen distribution and antimicrobial susceptibility patterns. Empirical antibiotic regimens should be periodically updated based on local antibiogram data to optimize neonatal sepsis management. Strengthening antimicrobial stewardship programs, enhancing infection prevention and control measures in neonatal intensive care units, and improving maternal health services are critical strategies to reduce neonatal infections. For a more comprehensive understanding of neonatal sepsis epidemiology and resistance dynamics in Ethiopia, future multicenter studies incorporating molecular diagnostics and characterization of resistance genes are recommended.

## Supporting information

S1 Dataset Minimal dataset underlying the findings of this study. The file contains the anonymized raw data used for all statistical analyses reported in the manuscript.
(XLSX)

## Abbreviations

ANC: Antenatal Care
AOR: Adjusted Odds Ratio
APGAR: Appearance, Pulse, Grimace, Activity, Respiration
ATRH: Asella Teaching and Referral Hospital
CI: Confidence Interval
CLSI: Clinical and Laboratory Standards Institute
COR: Crude Odds Ratio
CPS: Culture-Proven/Positive Sepsis
CRAB: Carbapenem-Resistant Acinetobacter baumannii
CRAB: Carbapenem-resistant Acinetobacter baumannii
CS: Clinical Sepsis
EDHS: Ethiopian Demographic Health Survey
EONS: Early-Onset Neonatal Sepsis
ESBL: Extended-Spectrum Beta-Lactamases
GBS: Group B *Streptococcus*
IMNCI: Integrated Management of Neonatal and Childhood Illness guideline
LONS: Late-Onset Neonatal Sepsis
MDR: Multidrug-Resistant
MRSA: Methicillin-Resistant *Staphylococcus aureus*
NICU: Neonatal Intensive Care Unit
PROM: Premature Rupture of Membrane(s)
WHO: World Health Organization.
